# Updating the Lamellar Hypothesis of Hippocampal Organization

**DOI:** 10.3389/fncir.2012.00102

**Published:** 2012-12-10

**Authors:** Robert S. Sloviter, Terje Lømo

**Affiliations:** ^1^Department of Neurobiology, Morehouse School of MedicineAtlanta, GA, USA; ^2^Department of Physiology, Institute of Basic Medical Sciences, University of OsloOslo, Norway

**Keywords:** hippocampus, hippocampal formation, dentate gyrus, entorhinal cortex, lamellar organization, lateral inhibition, mossy cells, inhibitory interneurons

## Abstract

Andersen et al. (1971) proposed that excitatory activity in the entorhinal cortex propagates topographically to the dentate gyrus, and on through a “trisynaptic circuit” lying within transverse hippocampal “slices” or “lamellae.” In this way, a relatively simple structure might mediate complex functions in a manner analogous to the way independent piano keys can produce a nearly infinite variety of unique outputs. The lamellar hypothesis derives primary support from the “lamellar” distribution of dentate granule cell axons (the mossy fibers), which innervate dentate hilar neurons and area CA3 pyramidal cells and interneurons within the confines of a thin transverse hippocampal segment. Following the initial formulation of the lamellar hypothesis, anatomical studies revealed that unlike granule cells, hilar mossy cells, CA3 pyramidal cells, and Layer II entorhinal cells all form axonal projections that are more divergent along the longitudinal axis than the clearly “lamellar” mossy fiber pathway. The existence of pathways with “translamellar” distribution patterns has been interpreted, incorrectly in our view, as justifying outright rejection of the lamellar hypothesis (Amaral and Witter, 1989). We suggest that the functional implications of longitudinally projecting axons depend not on whether they exist, but on what they do. The observation that focal granule cell layer discharges normally inhibit, rather than excite, distant granule cells suggests that longitudinal axons in the dentate gyrus may mediate “lateral” inhibition and define lamellar function, rather than undermine it. In this review, we attempt a reconsideration of the evidence that most directly impacts the physiological concept of hippocampal lamellar organization.

## Origins of the Lamellar Hypothesis

The anatomical features of the mammalian hippocampal formation are well described, and the importance of the hippocampus to memory formation and spatial navigation is undisputed, but exactly how the three-dimensional structural organization of the hippocampal formation governs its behavior at the network level, and how a relatively simple structure differentiates and encodes so many distinct memories and locations remains incompletely understood (O’Keefe and Nadel, 1978; Treves and Rolls, 1992; McNaughton et al., 1996; Leutgeb et al., 2007; Morris, 2007; Rolls, 2010). The lamellar hypothesis of hippocampal function in its simplest original form posited that excitatory activity travels from the entorhinal cortex and through the hippocampus via a “trisynaptic circuit” lying within a series of parallel hippocampal “slices” or “lamellae” (Andersen et al., 1969, 1971). In this way, it was envisaged that temporal lobe interactions between the entorhinal cortex and the hippocampus were organized topographically, and that “lamellae” might operate independently, permitting a relatively simple structure to mediate complex behaviors.

The lamellar hypothesis as originally conceived (Andersen et al., 1969) was greatly influenced by the still unpublished anatomical findings of Blackstad and his colleagues in Århus, Denmark, who had made two observations based on the distribution of degenerating fibers after focal injury in the dentate gyrus or entorhinal cortex. First, Blackstad et al. (1970) reported that after small lesions of the dentate gyrus, degenerating mossy fibers exhibited a “lamellar” pattern in the transverse plane, and they also noted that “very narrow bands were seen in a few animals with particularly small lesions.” Second, Andersen and colleagues cited as a personal communication from Jeune the subsequently published finding that, “each specific level of the entorhinal area distributes fibers to a restricted segment of the hippocampus” (Hjorth-Simonsen and Jeune, 1972). On the basis of these anatomical features, and the electrophysiological responses to afferent stimulation (Lømo, 1971), Andersen and colleagues suggested that entorhinal neurons topographically excite a thin strip of granule cells, which then topographically excites CA3 neurons, and so on, through the serial elements of the trisynaptic pathway lying within a transverse hippocampal “slice” (Andersen et al., 1969, 1971; Lømo, 1971). Unsurprisingly, anatomical studies have clearly demonstrated that the structural organization of the hippocampal trisynaptic circuit is far more intricate than originally appreciated.

The original lamellar hypothesis (Andersen et al., 1971) did not anticipate all of the implications of the longitudinal axonal distributions of Layer II entorhinal neurons, dentate hilar mossy cells and CA3 pyramidal cells, and because these structural features can be interpreted as being either consistent (Andersen et al., 2000; Lømo, 2009) or inconsistent (Amaral and Witter, 1989) with the lamellar hypothesis, we believe that the hypothesis merits discussion and updating in a way that takes all of the relevant information into account. In the original publication that introduced the lamellar hypothesis, Andersen et al. (1971) did not state or imply that lamellae are spatially rigid “hardware” units that function independently under all conditions, or that excitatory neurotransmission through the “trisynaptic circuit” must remain wholly within individual transverse “slices.” To the contrary, Andersen and colleagues stated that, *“the functional independence of neighboring lamellae suggests that the hippocampus, despite its stereotyped structure, may be capable of considerable operational flexibility,”* and noted that, *“the total output from the hippocampus would derive from a series of lamellae, the number and size of which would largely depend on the pattern of afferent impulses passing along the perforant path fibers from the entorhinal area.”* Perhaps most importantly, Andersen and colleagues also wrote that they had not directly examined, *“…the possible defocusing effect of the longitudinal associational fibers, which are collaterals of CA3 cells running parallel to the long axis of the hippocampus and making synaptic contact with other CA3 cells (Lorente De Nó, 1934).”* Thus, Andersen and colleagues proposed the lamellar hypothesis with the recognition and understanding that the longitudinal CA3 pathway existed, and that translamellar facilitation and inhibition mediated by longitudinal excitatory and inhibitory pathways would sculpt excitatory signals and govern the parameters of lamellar function (Andersen et al., 1971).

Following the earliest anatomical studies cited by Andersen and colleagues in their initial proposal of the lamellar hypothesis, tracer studies concluded that the entorhinal cortex forms a topographic, but somewhat divergent innervation of the dentate gyrus (Wyss, 1981; Ruth et al., 1982, 1988; Witter et al., 1989), and that both CA3 pyramidal cells (Lorente De Nó, 1934; Swanson et al., 1981) and dentate hilar neurons (Zimmer, 1971; Swanson et al., 1978; Berger et al., 1981; Laurberg and Sørensen, 1981) also form extensive longitudinal associational axonal projections. The relevance of longitudinally extensive afferent and associational pathways to the concept of lamellar organization was addressed in a commentary article by Amaral and Witter (1989), who concluded that the existence of pathways that travel in the septo-temporal plane was incompatible with *“a strict interpretation of the lamellar hypothesis,”* and stated that, *“clinging to the lamellar concept of hippocampal function is fast becoming detrimental to further advances in understanding structure/function relationships in this system.”* The outright rejection by Amaral and Witter of a “strict” version of the lamellar hypothesis that was neither stated nor implied by the original hypothesis (Andersen et al., 2000) has been so influential that only limited discussion of the lamellar hypothesis has subsequently appeared, and virtually no mention of the hypothesis is made in the recently published encyclopedic compendium of all things hippocampal (Andersen et al., 2007).

## Revisiting the Lamellar Hypothesis

In our view, the lamellar hypothesis has much to recommend it, and its appeal involves no attraction to outdated or obsolete concepts. To the contrary, we think it would be imprudent to discard a useful hypothesis unless its value has been irretrievably diminished. We suggest that the significance of longitudinally projecting axons depends not on whether they exist, but on what they do, and that the data from tracing studies can be just as readily interpreted as supporting the lamellar hypothesis as undermining it. In this review, we attempt a reconsideration of the evidence that most directly impacts the lamellar hypothesis. Our position is not that the hypothesis as originally formulated anticipated all subsequent findings, or that it should remain unmodified. Rather, we suggest that a reappraisal of all of the relevant data is warranted and compelling, and that the implications of the lamellar hypothesis for understanding hippocampal function need to be reconsidered in light of several issues that have not informed previous discussions of the subject.

The structural organization and function of the hippocampus can be viewed from different perspectives, and we do not pretend to know how to determine objectively which perspective might most closely approximate the truth. From the most fundamental biological article of faith that structure governs function, we assume that hippocampal “lamellar” function, if it is an operative physiological process that can be defined, is established and governed by the three-dimensional organization of the hippocampal formation, and also by other brain regions that influence hippocampal events. Before considering the connectivity of each hippocampal cell population in greater detail, we will examine the entorhinal input to the dentate gyrus by perforant path fibers because hippocampal information flow apparently starts there, and our understanding of how information in the entorhinal cortex reaches the hippocampus will facilitate an understanding of what the dentate gyrus might do and how it might do it.

### How “lamellar” or “non-lamellar” is the entorhinal cortex input to the dentate gyrus?

**Figure 1:**
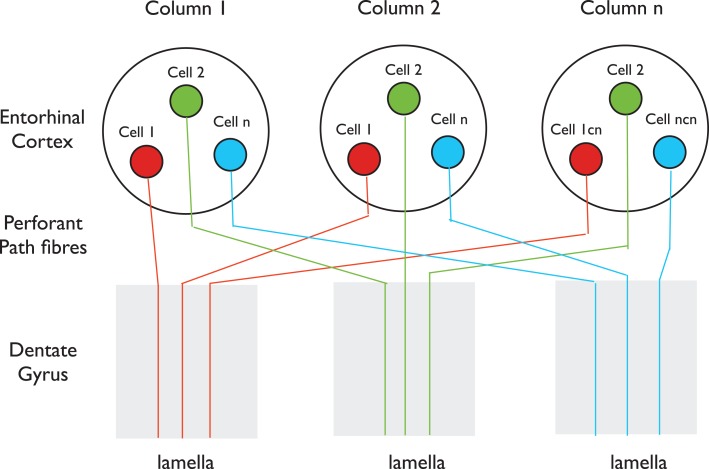
**A possible organization of the entorhinal input to the dentate gyrus**. The entorhinal cortex in this perspective consists of a mosaic of vertical columns. Each column contains subsets of cells of similar properties (red, green, or blue) present in each column. Cells in each subset project their perforant path axons into the same lamella. Tracers injected locally into either the entorhinal cortex or the dentate gyrus, after anterograde or retrograde transport, respectively, will label extensive regions of the dentate gyrus or entorhinal cortex, even though individual axons enter only one lamella. This scenario is consistent with both the longitudinally restricted axonal projections of single Layer II cells described by Tamamaki and Nojyo (1993), the divergent spread of tracers after focal injection into the entorhinal cortex (Witter, 2007) and the lamellar excitation of dentate granule cells by stimulation of perforant path fibers where they enter the dentate gyrus (Lømo, 2009).

A primary piece of anatomical evidence supporting the initial formulation of the lamellar hypothesis was the finding that the entorhinal fibers, *“…spread out in the septo-temporal direction…”* and that, *“each specific level of the entorhinal area distributes fibers to a restricted segment of the hippocampus”* (Hjorth-Simonsen and Jeune, 1972). The longitudinal spread of entorhinal fibers was understandable within a “lamellar” context, as noted by Hjorth-Simonsen and Jeune, because the entorhinal cortex is smaller than the hippocampus, and therefore, the outputs must fan out naturally to some extent to form a topographical “lamellar” innervation of the larger hippocampus. However, numerous studies using tracer injections reported that although the entorhinal cortex input to the dentate gyrus has clear topographic features, with different parts of the entorhinal cortex projecting to different segments of the dentate gyrus along the septo-temporal axis, it is nonetheless more divergent longitudinally than expected for a “lamellar” input (Ruth et al., 1982, 1988; Witter et al., 1989; Dolorfo and Amaral, 1998; van Groen et al., 2003; Witter, 2007). The conclusion that entorhinal cells form longitudinally extensive axonal terminations in the molecular layers of the dentate gyrus derives mainly from the observation that when an anterograde tracer is injected into the entorhinal cortex, the tracer is subsequently found to distribute along a significant expanse of the dentate gyrus (Witter, 2007). This finding seems to contradict the argument made by Andersen et al. (1971) that *“a point source of entorhinal activity projects its impulses… along a slice, or lamella, of hippocampal tissue.”* The tracer data appear similarly inconsistent with the observation that local stimulation of perforant path fibers near their entry to the dentate gyrus excites granule cells within a narrow transverse plane of the dentate gyrus, with the extracellular EPSP falling off sharply on either side (Lømo, 1971, 2009). However, no inconsistency arises if the entorhinal cortex contains separate columns that process incoming information similarly across the region, as do columns for individual parts of the visual field, the body surface, or composite sounds in primary visual, somatosensory, or auditory cortices (Kandel et al., 2000). In this scenario, co-mingled neurons that send longitudinally restricted axons to different portions of the dentate gyrus would appear, after injection of anterograde tracer into one site, to form highly divergent projections. Therefore, we hypothesize that cells in one such column (represented by Cell 1 in Figure 1) project their axons to one transverse lamella, while neighboring cells in the same column (Cell 2) project their axons to a different lamella, and so on for other neurons (Cell n) within the column. In addition, we suggest that cells with properties similar to Cell 1 in Columns 2 and 3 project their axons into the same lamella as Cell 1 in Column 1, and that cells with properties similar to Cell 2 project into the lamella of Cell 2, and so on (Figure 1). With such an organization, the results of both the tracer studies and the electrophysiological studies would be completely compatible. There is anatomical evidence that the entorhinal cortex, like other neocortical regions, is organized into a mosaic of similar columns (Witter and Moser, 2006). Intermingling of neurons that target spatially separated targets occurs in other parts of the cortex, for example in the primary motor cortex where adjacent neurons project to motor neurons that innervate different muscles (Andersen et al., 1975), and would give the appearance, after focal tracer injection, that all cells have divergent axonal projections.

### How divergent are the axons of Layer II entorhinal neurons along the septo-temporal axis?

Uncertainty regarding the extent of divergence of perforant path fiber input to the dentate gyrus makes two studies particularly relevant. First, if most or all Layer II entorhinal cortex neurons innervate wide expanses of the dentate gyrus, then two different retrograde tracers injected into two widely separated segments of the dentate gyrus should be transported and co-localized by most or all Layer II cells. This study was performed in the monkey, and although some co-localization of tracer was noted in some cells in the transitional area between the two cortical regions that project to the two tracer injection sites, individual cells containing both tracers were relatively rare (Witter et al., 1989). This finding suggests that most entorhinal neurons probably do not innervate a large expanse of the dentate molecular layer.

Second, if a small injection of anterograde tracer into the entorhinal cortex can label a broad expanse of the dentate gyrus because individual Layer II entorhinal neurons reliably innervate that same longitudinal expanse, then individual entorhinal neurons filled with dye should exhibit axons that span that large expanse. However, that does not appear to be the case (Tamamaki and Nojyo, 1993). Although reviews emphasizing the divergence of the entorhinal projection to the dentate gyrus cite 2.0 mm (∼20% of the length of the rat dentate gyrus) as the approximate longitudinal extent of the terminal arbors of individual Layer II entorhinal neuron axons (Amaral and Lavenex, 2007; Witter, 2007), we note the following points in the study by Tamamaki and Nojyo (1993), in which the axons of six individual Layer II neurons were described. Five of the six cells analyzed exhibited axons that extended <1.5 mm along the longitudinal axis, and the sixth cell did not reach 2.0 mm in its longitudinal extent. The average longitudinal spread of single axon terminals may therefore be <1.5 mm. Functionally, the width of the transverse strip, the size of the cluster of granule cells, or the spatial extent of the granule cells brought to firing by perforant path input in the normal state may be narrower still. This is likely to occur predominantly along the midline of the axon’s terminal field because that is where the efficiency or density of terminals is probably highest. That is also where EPSPs will summate most effectively and where repetitive impulse activity will cause the greatest “frequency potentiation,” as observed during repetitive stimulation at 10–20 Hz (Andersen et al., 1966; Bliss and Lømo, 1973), which corresponds to frequencies recorded *in vivo* from entorhinal cortex layer II cells projecting to the dentate gyrus during exploration (Fyhn et al., 2007).

Several methodological factors make it difficult to be certain about the axonal distribution of the six cells described by Tamamaki and Nojyo (1993). Of the 16 cells that were initially labeled, 10 cells were too inadequately filled for the authors to give any description of their axonal distribution. Thus, it remains a possibility that the remaining six cells that were reconstructed and described may have been only partially filled, and the longitudinal extent of their axonal projections underestimated, although the extensive cell-filling in the transverse plane suggests that the longitudinal axon distribution estimates of most cells may well be accurate (∼1.4 mm). These authors also stated that the somata of all six Layer II cells were from a dorsolateral subregion of the entorhinal cortex that specifically innervates the septal dentate gyrus, and that technical factors may have biased cell selection for large neurons. Therefore, these six cells may be a non-representative subset of all Layer II neurons (Tamamaki and Nojyo, 1993).

Thus, we are left with uncertainty about several anatomical features that have unknown significance for the functional issue being discussed. But regardless of whether an “anatomical lamella” or granule cell “cluster” is only as wide as the narrowest part of the mossy fiber pathway (∼0.2 mm), or is the width of the entorhinal input to the dentate gyrus and most inhibitory interneurons (∼1.0–1.5 mm), we do not think that these anatomical features can be assumed to have linear control of lamellar physiology. That is, we do not see the transverse strips or clusters of active granule cells as “hard-wired” anatomical lamellae of a fixed width, but rather, as narrow functional strips or clusters of activity, one strip or cluster flowing into others, shifting position and width as events unfold, and interacting via longitudinal connections. Assuming that most entorhinal neurons have a ∼1.0–1.4 mm axonal divergence along the septo-temporal axis, the available data support the lamellar hypothesis in the sense that individual entorhinal neurons apparently send their axons to a relatively narrow transverse segment of the dentate gyrus (Tamamaki and Nojyo, 1993), as originally stated by Hjorth-Simonsen and Jeune (1972).

Although the available anatomical data can be cited to support opposite perspectives, and the scheme in Figure 1 above may be wrong, it is nonetheless difficult to reconcile the observed activation of a narrow transverse strip of dentate granule cells in the rabbit (Lømo, 1971, 2009), or the idea of pattern separation (McNaughton, 1989; Leutgeb et al., 2007), with the idea of a uniformly diffuse non-lamellar input to the dentate gyrus. Clearly, should future studies demonstrate that most or all Layer II neurons possess axons that target granule cells along most or all of the dorsal dentate gyrus (∼4.0 mm), as concluded by Amaral and Witter (1989), our suggested scheme would be incorrect. Regardless, the issue of the longitudinal divergence of the entorhinal input to the dentate gyrus may be a moot point with regard to lamellar function because it is clear that even if the entorhinal input to the dentate gyrus is only 1.0–1.5 mm along the longitudinal axis (Tamamaki and Nojyo, 1993), it is still more extensive longitudinally than the clearly “lamellar” mossy fiber pathway, which may be as limited as ∼0.2 mm in the septo-temporal plane (Blackstad et al., 1970; Claiborne et al., 1986).

### Functional implications of a possibly divergent entorhinal input to the dentate gyrus

How does the recognition that the entorhinal input to the dentate gyrus is more extensive longitudinally than the mossy fiber pathway impact the concept of lamellar function? Very little, in our view, for several reasons. First, since we have argued that the existence of longitudinal associational pathways is not, in and of itself, evidence against the concept of lamellar function, we view the anatomical features of the entorhinal input to the hippocampus similarly. That is, we would argue that the existence of a somewhat divergent afferent pathway has no obvious implications for lamellar function because the physiological implications of anatomical features cannot be easily inferred. Second, it is now clear that the perforant path input to the dentate gyrus directly innervates molecular layer dendrites of granule cells (McNaughton et al., 1981), basket cells, and axo-axonic cells (Soriano and Frotscher, 1989; Zipp et al., 1989), as well as the dendrites of hilar mossy cells (Figure 7C of Sloviter, 1983) and hilar somatostatin-positive neurons that send dendrites into the molecular layer (Sloviter, 1983; Leranth et al., 1990; Soltesz et al., 1993; Frotscher et al., 1994; Buckmaster, 2012). Thus, the perforant path input to the dentate gyrus could evoke feed-forward inhibition in granule cells (Buzsáki, 1984; Sloviter, 1991a), thereby inhibiting weakly excited granule cells and resulting in a highly focused excitation of granule cells, perhaps at the center of a somewhat divergent entorhinal axonal plexus. In addition, strong recurrent inhibition of dentate granule cells, as demonstrated in the rabbit (Lømo, 2009), might similarly “sharpen” the input message, as might an inhibitory component included in the entorhinal input to the dentate gyrus (Germroth et al., 1989). Regardless, it seems highly doubtful that the net physiological influence of any axonal pathway can be inferred solely, or even secondarily, from its structural dimensions.

## The Longitudinal Extent of the Associational Axons of Hippocampal Neurons: An Overview

Before discussing the features of the different hippocampal neuron subpopulations, we summarize here (Table 1) the main hippocampal cell types with reference to the relative extents of their longitudinal associational axon projections, because the existence of these projections has been cited as the primary evidence undermining the concept of lamellar function (Amaral and Witter, 1989).

**Table 1:** **Comparative lengths of longitudinal associational axon projections of different hippocampal neuron subpopulations**.

Cell type	Axon plexus longitudinal length	Reference
Entorhinal layer II pyramidal neurons	∼1.4–1.9 mm	Tamamaki and Nojyo (1993)
Dentate granule cells^+^	∼0.2–1.3 mm	Blackstad et al. (1970), Claiborne et al. (1986), Acsády et al. (1998), and Ropireddy and Ascoli (2011)
Dentate mossy cells [periodicity of axonal distribution (Soltesz et al., 1993) : ∼900 μm]	∼6.6 mm	Amaral and Witter (1989), Soltesz et al. (1993), and Buckmaster et al. (1996)
Dentate basket cells	∼1.0–1.5 mm	Struble et al. (1978), Amaral and Lavenex (2007), Freund and Buzsáki (1996), and Sík et al. (1997)
Dentate hilar dendritically projecting cells^#^	∼1.0–1.5 mm	Buckmaster and Schwartzkroin (1995), Freund and Buzsáki (1996), and Sík et al. (1997)
CA3 pyramidal cells [periodicity of axonal distribution and HSP72 expression after ischemic injury (Hsu and Buzsáki, 1993; Li et al., 1994) : ∼300–600 μm]	∼4–7 mm	Ishizuka et al. (1990), Li et al. (1994), and Tamamaki and Nojyo (1991)
CA1 pyramidal cells (associational fibers)	Negligible	Amaral et al. (1991) and Amaral and Lavenex (2007)

*^+^Note that the longitudinal course taken by the mossy fibers at the end of their trajectory at particular septo-temporal levels (Swanson et al., 1978; Tamamaki and Nojyo, 1991; Acsády et al., 1998; Ropireddy and Ascoli, 2011) is not considered here*.

*^#^Note that the distances are those in which most axon length is sequestered, not the longest distance traversed by a single fiber*.

Hippocampal neurons with longitudinally limited associational axon projections, i.e., neurons that form few associational axons, or keep most of their axons within ∼1.0–1.5 mm of their somata, include dentate granule cells (Blackstad et al., 1970; Gaarskjaer, 1978, 1981), most or virtually all hippocampal inhibitory interneurons (Struble et al., 1978; Buckmaster and Schwartzkroin, 1995; Freund and Buzsáki, 1996; Sík et al., 1997; Zappone and Sloviter, 2001, 2004; Gloveli et al., 2005; Amaral and Lavenex, 2007), and CA1 pyramidal cells, which form longitudinal projections to the subiculum, but few associational axons that interconnect CA1 pyramidal cells (Amaral et al., 1991; Amaral and Lavenex, 2007). Hippocampal neurons with extensive longitudinal associational axons (most axon length greater than ∼1.5 mm from the soma of origin) include only dentate hilar mossy cells (Soltesz et al., 1993; Buckmaster et al., 1996) and CA3 pyramidal cells (Ishizuka et al., 1990; Li et al., 1994). It should be noted that although hippocampal interneurons restrict most of their ipsilateral associational axon length to within ∼1 mm of their somata, many of the same interneurons form long-axon projections that innervate the contralateral hippocampus and the medial septum (Zappone and Sloviter, 2001, 2004).

In the discussion that follows, we address the question of whether the available anatomical evidence really negates the lamellar hypothesis, or whether longitudinal “translamellar” axons could be consistent with a model in which the granule cells are functionally separated from adjacent granule cells by lateral inhibition, and further, whether granule cell information transmitted to mossy cells and CA3 pyramidal cells via the undisputedly “lamellar” mossy fiber pathway is then conveyed topographically to targets at multiple levels throughout the longitudinal axis of the hippocampus, inhibiting some targets and exciting others. In this scenario, the net effect of “translamellar” axonal projections would depend on the net effects of longitudinally projecting mossy cells and CA3 pyramidal cells on principal cells or interneurons at different levels along their longitudinal trajectories (Gulyás et al., 1993; Sik et al., 1993; Bernard and Wheal, 1994; Wittner et al., 2006; Ropireddy et al., 2011). If these effects include excitation of distant inhibitory neurons, then the existence of longitudinally projecting axons could define lamellar function, rather than undermine it. After all, how could activity in spatially separated “lamellae” or “clusters” of pyramidal cells or granule cells be coordinated (Deadwyler and Hampson, 1999; Hampson et al., 1999; Small, 2002) without the involvement of longitudinal excitatory projections?

## Three-Dimensional Organization of the Dentate Gyrus

### Is the existence of longitudinally projecting mossy cell axons necessarily antithetical to the concept of lamellar function?

It is clear and undisputed that whereas the dentate granule cells send “lamellar” axons to their hilar and area CA3 target cells (Blackstad et al., 1970; Gaarskjaer, 1978, 1981; Claiborne et al., 1986; Acsády et al., 1998; Ropireddy and Ascoli, 2011), dentate hilar mossy cells form a complementary longitudinal axon system that preferentially innervates distant segments of the dentate gyrus along the septo-temporal axis. That is, mossy cells preferentially avoid innervating the inner molecular layer within the “lamella” in which they receive mossy fiber excitation, but instead, apparently innervate local interneurons in the hilus of their “home” lamella before their axons enter the molecular layer and travel longitudinally to preferentially innervate the inner molecular layer of distant segments of the dentate gyrus (Amaral and Witter, 1989; Soltesz et al., 1993; Buckmaster et al., 1996). Importantly, whereas the convergence of granule cell input to inhibitory interneurons is high, convergence of mossy fibers onto mossy cells and CA3 pyramidal cells is low (Acsády et al., 1998), which is a structural feature consistent with a highly topographic (“lamellar”) excitation of targeted principal cells by individual granule cells. That is, many granule cells contribute to the convergent activation of nearby inhibitory interneurons that presumably generates intralamellar inhibition, whereas the non-convergent innervation of mossy cells and CA3 pyramidal cells likely conserves the point-to-point lamellar nature of the transmission from granule cells to their excitatory target cells.

The “lamellar” pattern of mossy fiber distribution and the complementary “translamellar” pattern of mossy cell axon distribution prompt several questions. First, what purpose does it serve for granule cells to severely limit the longitudinal spread of their initial communication if that highly focused message is simply going to be extensively amplified and spread longitudinally to other granule cells by mossy cells, thereby “defocusing” the excitation of the initially targeted lamella by the entorhinal input? Second, is the existence of excitatory longitudinal associational fibers, in and of itself, antithetical to the concept of lamellar organization? The answers to these questions are apparent, but only if lamellar function is defined by “on-beam” excitation of granule cell target cells and “off-beam” translamellar lateral inhibition that spatially restricts granule cell excitation (Sloviter, 1994; Zappone and Sloviter, 2004). This hypothesized organization within the dentate gyrus may have significant similarities to the structural organization of the cerebellum, in which “on-beam” excitation of Purkinje cells by the parallel fibers is focused by lateral inhibition mediated via excitation of inhibitory interneurons (Eccles et al., 1967; Ito, 1984; Cohen and Yarom, 2000; Gao et al., 2006). Thus, if the mossy cell-derived longitudinal axon system activates distant dentate inhibitory interneurons, thereby producing lateral granule cell inhibition in distant granule cells, the “lamellar” pattern of granule cell axon distribution and the “translamellar” pattern of mossy cell axon distribution might establish lamellar function, rather than undermine it. Similarly, if two groups of spatially separated CA1 pyramidal cells must discharge synchronously to encode a particular memory or recognize a particular location in space (Deadwyler and Hampson, 1999; Hampson et al., 1999), transverse and longitudinal axon collaterals of CA3 pyramidal cells may be the primary means of synchronizing two or more spatially separated target populations. Of particular relevance to this issue are reports that both dentate mossy cells and CA3 pyramidal cells innervate their respective target regions unevenly, with periodic clusters of axon length occurring at intervals of ∼900 μm for mossy cells (Soltesz et al., 1993) and ∼300–600 μm for CA3 pyramidal cells (Hsu and Buzsáki, 1993; Li et al., 1994), respectively. This pattern of axon distribution by excitatory longitudinal fibers, which might form “bands” of excitation and inhibition via excitation of principal cells and inhibitory interneurons (Wittner et al., 2006), respectively, may be entirely consistent with lamellar organization (Li et al., 1994). That is, communication between spatially separated neurons or neuronal clusters (Deadwyler and Hampson, 1999; Hampson et al., 1999; Small, 2002) would require an excitatory longitudinal associational pathway to coordinate this spatially separated activity.

### Lateral inhibition in the dentate gyrus as a mechanism defining lamellar function

The anatomical perspective that the “translamellar” distribution pattern of dentate mossy cell axons is, in and of itself, antithetical to the concept of lamellar function (Amaral and Witter, 1989) was the logical consequence of assuming that hilar mossy cells directly excite distant granule cells, thereby defocusing the entorhinal excitation of the granule cell layer. However, this does not appear to be the case. In studies performed *in vivo* before the features of the ipsilateral longitudinal associational projections of mossy cells were fully appreciated, Buzsáki and colleagues (Buzsáki and Czeh, 1981; Buzsáki and Eidelberg, 1981, 1982) and Goddard and colleagues (Douglas et al., 1983; Bilkey and Goddard, 1987) reported that stimulation of the excitatory dentate commissural pathway formed by glutamatergic mossy cells (Frotscher, 1992; Soriano and Frotscher, 1994) produced a paradoxical net inhibitory effect on contralateral granule cells because the commissural excitation of basket cells apparently predominated over “very weak” commissural excitation of granule cells (Douglas et al., 1983). The subsequent finding that mossy cells also form an extensive associational axon collateral system (Amaral and Witter, 1989) should have led to the hypothesis that mossy cells might also excite basket cells ipsilaterally, producing granule cell lateral inhibition, rather than lateral excitation, but that connection was not made at the time.

It was the discovery that the seizure-induced death of hilar mossy cells is closely associated with the immediate development of granule cell hyperexcitability (Sloviter, 1987, 1991b), taken together with the data indicating the translamellar pattern of mossy cell axon distribution (Amaral and Witter, 1989), that led us to propose the hypothesis that seizure-induced mossy cell death might denervate distant basket cells, resulting in translamellar granule cell disinhibition (Sloviter, 1994). The concept of translamellar granule cell disinhibition implied the existence of normal translamellar inhibition, and this implication led us to determine whether lateral inhibition exists in the normal dentate gyrus (Sloviter and Brisman, 1995), and whether it is abolished following extensive hilar neuron loss (Zappone and Sloviter, 2004).

To selectively activate a “lamella” or “cluster” of granule cells, and then determine the effect of that discharge on granule cell responses in a distant “lamella,” is not a trivial undertaking because even highly localized electrical stimulation of the granule cell layer might produce ipsilateral effects a few millimeters along the longitudinal axis via unidentifiable pathways or mechanisms (Hetherington et al., 1994). Therefore, we developed a method of locally delivering the GABA-A receptor antagonist bicuculline to the granule cell layer to produce a highly localized and augmented granule cell layer discharge in response to afferent stimulation outside the hippocampus. During continuous perforant path stimulation *in vivo* at 0.3 Hz, passive diffusion of bicuculline methiodide from the tip of a recording microelectrode resulted in spatially restricted (<1 mm) granule cell layer discharges that caused powerful, long-lasting (>150 ms) lateral inhibition at distant granule cell layer recording sites up to 4.5 mm along the septo-temporal axis (Sloviter and Brisman, 1995; Zappone and Sloviter, 2004). Thus, “lamellar” granule cell layer discharges produce powerful distant granule cell lateral inhibition, which apparently predominates over an underlying “associative” excitation of granule cells by mossy cells (Zappone and Sloviter, 2004).

Importantly, only *extensive* hilar neuron loss caused by prolonged perforant path stimulation abolished this translamellar inhibitory effect (Zappone and Sloviter, 2004). Minor mossy cell loss in kainate-treated rats (Zappone and Sloviter, 2004), or in hippocampal slices in which a small percentage of mossy cells were manually destroyed after being visually identified (Ratzliff et al., 2004), failed to replicate the granule cell hyperexcitability associated with extensive hilar neuron loss. The hypothesis that granule cell hyperexcitability is specifically caused by *extensive* mossy cell loss (Sloviter, 1994; Zappone and Sloviter, 2004) is supported by a recent study in a conditional knockout mouse that selectively expresses the diphtheria toxin receptor in hilar mossy cells. In these animals, diphtheria toxin triggers selective and extensive mossy cell loss, immediate granule cell hyperexcitability *in vitro*, as well as impaired pattern separation in the dentate gyrus (Jinde et al., in press).

Consistent with the studies in rats described above, strong stimulation of perforant path fibers where they enter the dentate gyrus causes dentate granule cells to discharge along a narrow transverse strip in the rabbit (Lømo, 2009). Stimulation of mossy fibers in area CA3 activates a similar transverse strip antidromically. Both activations are accompanied immediately afterward by lateral inhibition, which in the rabbit lasts up to 100 ms and spreads 4–5 mm to either side of the strip (Lømo, 2009). Evidently, impulses along collaterals of granule cell axons activate local interneurons (Acsády et al., 1998) that inhibit granule cells. Local inhibitory interneurons likely cause the inhibition within and immediately outside the lamella, whereas mossy cells may inhibit granule cells at greater longitudinal distances via excitation of inhibitory interneurons, as described in the preceding paragraphs. Signs of feed-forward inhibition did not appear under the conditions of these experiments (Lømo, 2009), but may well occur given that many inhibitory interneurons extend dendrites into the molecular layer, and may respond to volleys along the perforant path before the granule cells discharge (Buzsáki, 1984).

### Longitudinal influences of dentate gyrus inhibitory interneurons

If individual lamellae of the dentate gyrus, spatially restricted clusters of granule cells, or individual granule cells function independently and form unique combinations of temporally associated activity with other granule cells in distant locations, then the inhibitory interneurons that mossy cells excite would be predicted to have longitudinally restricted axons that inhibit individual lamellae, rather than expansive axonal networks that inhibit large segments of the granule cell layer. That is, if different combinations of spatially separated granule cells need to discharge in unison under different conditions, it would be hypothetically unproductive for the activation of cells in one lamella to inhibit all other lamellae indiscriminately. Thus, mossy cell activation of distant interneurons having longitudinally restricted axonal projections might ideally permit control of individual lamellae, allowing distantly separated neurons to discharge in varying combinations, perhaps to encode specific memories (Small, 2002) or to register, remember, and locate particular spatial locations (Deadwyler and Hampson, 1999; Hampson et al., 1999).

The results of virtually all studies of the longitudinal axonal distributions of dye-filled and reconstructed interneurons appear consistent with lamellar organization because virtually all interneurons studied keep most of their axon length within ∼1 mm of their somata (Amaral and Lavenex, 2007). Although the method of filling individual neurons with a dye and then calculating the longitudinal extent of their axons from reconstructed consecutive sections is technically difficult and prone to underestimation when a cell is incompletely filled (Sík et al., 1997), the studies of the axonal fields of the few dentate gyrus interneurons that have been filled and reconstructed have consistently shown that most of the axon length of inhibitory interneurons remains close to the soma, with a sharp drop-off of axon length as the septo-temporal distance from the soma increases past ∼1 mm (Buckmaster and Schwartzkroin, 1995; Sík et al., 1997). Filled hilar interneurons that innervate the perforant path termination zone in the outer dentate molecular layer, and correspond to the hilar somatostatin-positive population (Bakst et al., 1986; Sloviter and Nilaver, 1987; Halasy and Somogyi, 1993), also concentrate most of their axonal length within their lamellae of origin (Amaral and Witter, 1989; Buckmaster and Schwartzkroin, 1995; Freund and Buzsáki, 1996; Sík et al., 1997), although a few axons extend farther (Buckmaster and Schwartzkroin, 1995), possibly on their way to the septum (Zappone and Sloviter, 2001). The few dentate basket cells that have been analyzed exhibit a similarly restricted axonal distribution (Sík et al., 1997), with axons extending ∼1.0–1.5 mm along the septo-temporal axis (Struble et al., 1978; Sík et al., 1997), which is, interestingly, the width of the septo-temporal expanse specifically avoided by the mossy cells (Amaral and Witter, 1989; Soltesz et al., 1993; Buckmaster et al., 1996).

The small body of data describing individual inhibitory interneurons that have been filled *in vivo* and reconstructed, and the complete lack of information about how generally extensive the longitudinal associational projections of most hippocampal interneuron subpopulations might be, led us to initiate retrograde tracer studies to identify the extent of both the commissurally- and associationally projecting interneuron subpopulations (Zappone and Sloviter, 2001, 2004). Although a retrograde tracer study cannot describe the projections of any given single cell, its strength lies in the ability of a tracer to label a large percentage of any neuronal population capable of transporting the tracer from a particular location. Thus, tracer studies give a more representative estimate of the extent of the axonal projections formed by entire cell populations, as opposed to the features of single cells that may not be representative of the larger cell population. The results of these retrograde tracer studies showed that a majority of dentate gyrus interneurons, and virtually all somatostatin-positive hilar interneurons, innervate the contralateral hippocampus, and that the somatostatin-positive interneurons of both the hippocampal stratum oriens and the dentate hilus are a unique population of long-axon-, septally projecting hippocampal interneurons (Zappone and Sloviter, 2001), a finding that has been confirmed by other laboratories (Jinno and Kosaka, 2002; Gulyás et al., 2003; Melzer et al., 2012; Quilichini et al., 2012). Most importantly, despite their ability to pick up and retrogradely transport tracer from the distant septum or the distant contralateral hippocampus, these same somatostatin-positive interneurons consistently failed to take up and transport the same tracer when it was placed only ∼2.5 mm along the septo-temporal axis (Zappone and Sloviter, 2004). This finding is consistent with the observations in filled cells that somatostatin-positive hilar interneurons concentrate their axons within their “home” lamellae (Buckmaster and Schwartzkroin, 1995; Sík et al., 1997; Amaral and Lavenex, 2007), and with the results of anterograde tracer distribution to the outer dentate molecular layer mentioned in a footnote in the study by Amaral and Witter (1989).

All known interneuron populations of the dentate gyrus showed similarly minimal associational transport of retrograde tracer (Zappone and Sloviter, 2001, 2004). Immediately adjacent to the tracer injection site, all interneurons with the morphologies and locations of dentate basket cells, molecular layer axo-axonic cells, and hilar dendritically projecting interneurons contained tracer in their somata, indicating that all interneuron subpopulations studied have the capacity to take up and retain the tracer locally. Although many parvalbumin-positive dentate basket cells readily transported tracer from the distant contralateral hippocampus (Goodman and Sloviter, 1992; Zappone and Sloviter, 2001), the same cells did not transport tracer placed only ∼2 mm along the septo-temporal axis (Zappone and Sloviter, 2004). Similarly, the population of axo-axonic interneurons of the dentate molecular layer that innervate the axon initial segments of granule cells (Soriano and Frotscher, 1989) appeared to be exclusively short-axon cells because they did not transport tracer from the contralateral or ipsilateral hippocampus, or from the septum (although the possibility of long projections to other areas not studied cannot be excluded). Thus, no interneuron subpopulation appeared to possess significant, longitudinally extensive associational axonal projections despite often having much longer commissural and septal axon projections (Zappone and Sloviter, 2001). These results using a retrograde tracer are consistent with the findings of the single cell studies indicating that the longitudinal axonal projections of hippocampal interneurons are rarely significant more than ∼1 mm from the soma in the septo-temporal direction (Struble et al., 1978; Buckmaster and Schwartzkroin, 1995; Freund and Buzsáki, 1996; Sík et al., 1997; Gloveli et al., 2005; Amaral and Lavenex, 2007).

The finding that focal granule cell discharges caused powerful and long-lasting lateral inhibition in longitudinally distant granule cells (Zappone and Sloviter, 2004), taken together with the anatomical data on the longitudinal axonal projections of each cell type cited above, has led us to suggest that: (1) granule cells excite hilar mossy cells and interneurons “on-beam” via the “lamellar” mossy fiber projection (Acsády et al., 1998), (2) this lamellar excitation produces monosynaptic intralamellar granule cell inhibition via direct mossy fiber excitation of dentate inhibitory neurons, and disynaptic intralamellar granule cell inhibition via mossy fiber excitation of mossy cells that then excite inhibitory neurons, and (3) excitation of longitudinally distant inhibitory interneurons by mossy cells evokes distant disynaptic translamellar lateral inhibition (Sloviter, 1991b, 1994; Zappone and Sloviter, 2004). These three processes might collectively restrict granule cell excitation to the lamella most powerfully targeted by an entorhinal input.

## Implications of the Lamellar Hypothesis for Understanding Dentate Gyrus Function and Malfunction

It should be clearly understood that we regard the lamellar hypothesis to be a general perspective regarding the functional implications of a number of structural features, rather than a specific and rigid proposal that all granule cells within a transverse plane must discharge in synchrony in response to a perforant path input that is anatomically extensive in the transverse plane (Tamamaki and Nojyo, 1993), or that all activity within the trisynaptic circuit must remain within the transverse plane.

In the freely behaving rat, specific behavioral tasks apparently involve the discharge of very few granule cells (Alme et al., 2010), whereas the large-amplitude population spikes evoked by angular bundle stimulation reflect a mass discharge that is undoubtedly an artifact of the experimental condition (Andersen et al., 1966; Lømo, 1971; McNaughton et al., 1981). Therefore, a physiological “lamellar” discharge could involve very few granule cells, and an activation of a correspondingly small number of CA3 pyramidal cells (Deguchi et al., 2011), as long as the longitudinal spread of the granule cell excitation is restricted. It is perhaps surprising that so few granule cells discharge during normal behavior (Alme et al., 2010) and at such low firing frequencies (<0.2 Hz; Jung and McNaughton, 1993). More recent studies, however, report that higher spike frequencies (averaging about 1 Hz) normally occur (Leutgeb et al., 2007; Mistry et al., 2011). Moreover, during behavioral tasks, many perforant path axons conduct impulses at rates of 10–20 Hz or more (Fyhn et al., 2007), while granule cells may generate high frequency bursts of impulses (Mistry et al., 2011). Interestingly, such bursts occur primarily on a background of very low mean frequencies, and only stimulus patterns that mimic this pattern are capable of inducing long-term potentiation (LTP) at mossy fiber synapses with CA3 pyramidal cells. The LTP at mossy fiber synapses is presynaptic and NMDA receptor-independent (non-Hebbian), whereas the LTP at perforant path-granule cells synapses is associative (Hebbian) and requires NMDA receptor activation, for example through some stronger coincident input. Perforant path synapses are generally weak because substantial summation of perforant path evoked EPSPs is needed in order to discharge granule cells (Lømo, 1971; McNaughton et al., 1981). Yet they undergo LTP if additional perforant path or other inputs evoke sufficient coincident depolarization. LTP at both perforant path and mossy fiber synapses are commonly induced in narrow, transverse slices of the hippocampus, consistent with the lamellar hypothesis proposed here, and with the idea that storage and recall of information from the entorhinal cortex require persistent changes in synaptic efficiency. For example, in mice in which dentate granule cells specifically lack NMDA receptors, LTP at perforant path – granule cell synapses (but not at other synapses in the hippocampus) is abolished, together with the ability to retain memories that allow discrimination between similar environments in ways that are consistent with pattern separation (McHugh et al., 2007).

In contrast to the normal behavioral state in which few granule cells apparently discharge in response to physiological afferent input, we suggest that it is entirely possible that many or all granule cells within a thin transverse plane might discharge synchronously when hilar neurons are extensively injured and granule cells become immediately disinhibited (Sloviter, 1991b, 1994). The recent observations that mature granule cells in normal rats are strongly inhibited, whereas newly born granule cells are unusually responsive because their axo-somatic inhibitory innervation is apparently still incomplete (Kempermann, 2012; Marin-Burgin et al., 2012), suggests that newly born, hyperexcitable granule cells constitute a disproportionate population of the granule cells that normally encode memories or locations, whereas mature granule cells are more inhibited and thereby perhaps held in “reserve” for specific tasks (Alme et al., 2010; Nakashiba et al., 2012). We suggest that the seizure-induced hilar neuron loss that produces immediate granule cell hyperexcitability (Sloviter, 1987, 1991b, 1994) may convert the inhibitory status of mature granule cells from “inhibited” to the “disinhibited” phenotype of newly born granule cells, thereby calling “reserve” granule cells to “active-duty.” This possible recapitulation of ontogeny as a result of a disinhibiting and epileptogenic hippocampal injury might explain why weak afferent stimulation in these injured animals evokes massive granule cell population spikes (Sloviter, 1991b, 1994). That is, inhibited mature granule cells that are normally resistant to discharging (Alme et al., 2010) presumably become disinhibited and join the younger, already disinhibited granule cells (Marin-Burgin et al., 2012) in their response to entorhinal input. In this instance, when young and older granule cells become similarly disinhibited, a lamellar discharge might involve a full strip of granule cells discharging synchronously within a thin transverse lamella, or in multiple adjacent lamellae (Sloviter, 1994). Thus, normal “lamellar” function might involve very few granule cells discharging in the normal state, whereas “lamellar” dysfunction might involve full strips of granule cells discharging in a spontaneously epileptic hippocampus (Sloviter, 1994; Bumanglag and Sloviter, 2008).

Normally, however, the dentate gyrus appears to perform pattern separation of inputs coming from the entorhinal cortex (McNaughton, 1989; Leutgeb et al., 2007; McHugh et al., 2007; Nakashiba et al., 2012). That is, patterns representing similar information are separated and the small differences between them augmented to facilitate their later recall as different. The CA3 region is then thought to perform pattern completion by storing the separated patterns in such a way that when a partial version of one of them is presented later, a more complete version of it can be reactivated. One recent computational model able to perform some form of pattern separation, storage, and recall divides dentate gyrus and CA3 into lamellae, exploits known properties of excitatory mossy cells and hilar inhibitory interneurons, and adds back-propagation from CA3 to inhibit granule cells within lamellae (Myers and Scharfman, 2011). However, the model does not incorporate the possibility that mossy cells activate basket cells more powerfully than granule cells (Douglas et al., 1983; Misgeld et al., 1992a,b; Scharfman, 1995), or that the perforant path input may also be functionally lamellar. Nor do most other published models of dentate-CA3 processing take these factors into account. As long as these are real possibilities, we believe that these features need to be considered if a more complete understanding of dentate gyrus and CA3 function is to be reached. In particular, if the perforant path splits information from the entorhinal area into multiple lamellar units, this may be one of the mechanisms by which pattern separation is established in the dentate gyrus.

## CA3 Pyramidal Cells and Their Extensive Associational Axons

In retrospect, it is easy to see how the longitudinal projections of dentate hilar mossy cells could have been viewed from a purely anatomical perspective as being antithetical to the idea of lamellar function (Amaral and Witter, 1989). Given the assumption that longitudinal excitatory axons of hilar mossy cells must excite distant granule cells (Amaral and Witter, 1989; Buckmaster and Schwartzkroin, 1994), despite it having never been demonstrated, it was possible to conclude that, *“…the extensive associational projections of the dentate gyrus have the potential of widely dispersing the inputs that come into any particular level”* (Witter et al., 1989). With regard to whether the excitation of CA3 and CA1 pyramidal cells by associational axonal projections of CA3 pyramidal cells should have been viewed as being similarly inconsistent with lamellar function (Amaral and Witter, 1989), it is interesting to note which cells are innervated or avoided by CA3 pyramidal cells. Like dentate granule cells, which only innervate ipsilateral hilar and area CA3 neurons, CA3 pyramidal cells have a limited number of targets. CA3 pyramidal cells form recurrent excitatory connections with other CA3 pyramidal cells (Miles and Wong, 1987), and also innervate CA1 pyramidal cells, axo-somatic inhibitory interneurons (Wittner et al., 2006), and the lateral septum (Swanson et al., 1981). CA3 pyramidal cells do not innervate the medial septum, subiculum, presubiculum, parasubiculum, or the entorhinal cortex (Amaral and Lavenex, 2007). Thus, CA3 pyramidal cells receive lamellar information from granule cells, and primarily convey that information topographically to other CA3 pyramidal cells, to axo-somatic inhibitory interneurons, and to CA1 pyramidal cells. CA1 pyramidal cells, in turn, topographically innervate the ipsilateral subiculum (Amaral et al., 1991). The topographic distribution of CA3 pyramidal cell axons (Ishizuka et al., 1990; Li et al., 1994), the selective innervation by CA3 pyramidal cells of the interneuron subpopulation that mediates powerful axo-somatic pyramidal cell inhibition (Wittner et al., 2006), and the lack of CA3 pyramidal cell innervation of the subiculum and entorhinal cortex, indicate that CA3 pyramidal cells primarily convey lamellar input information within the hippocampus “proper,” and do not directly influence the entorhinal source of hippocampal input. Although CA3 pyramidal cell axons clearly do not restrict their axons to a transverse lamella, as acknowledged by Andersen et al. (1971), *the collective picture of the trisynaptic circuit, from our perspective, speaks to the preservation of topographic lamellar information within the trisynaptic circuit*.

## Possible “Lamellar” Influences of Septal and Brainstem Inputs to Inhibitory Interneurons

The perspective that the mere existence of longitudinal pathways undermines the entire concept of lamellar function (Amaral and Witter, 1989) ignores the possible involvement of other influences that might impose lamellar function on a structure that includes longitudinal circuitry. Several external inputs to the dentate gyrus and hippocampus “proper” might serve such a role. A prominent reciprocal relationship exists between the hippocampus and the medial septum/diagonal band of Broca (MSDB; Buzsáki et al., 1981; Nyakas et al., 1987; Freund and Antal, 1988; Leranth and Frotscher, 1989; Bland and Oddie, 1998; Takács et al., 2008). Virtually all hippocampal interneurons that innervate the septum belong to a single subpopulation of hippocampal interneurons: the somatostatin-positive cells of both the dentate gyrus and the hippocampus “proper” (Zappone and Sloviter, 2001, 2004). These hippocampal septally projecting (HS) cells directly innervate GABAergic and cholinergic neurons of the MSDB (Tóth et al., 1993; Gulyás et al., 2003; Takács et al., 2008). Thus, dentate hilar somatostatin-positive interneurons, which receive highly “lamellar” information from the granule cells, convey this topographically derived information directly to the medial septum. In turn, the cholinergic and GABAergic neurons of the septum/MSDB innervate hippocampal mossy cells, basket cells, and the somatostatin-positive hippocampo-septal cells (Freund and Antal, 1988; Freund and Buzsáki, 1996; Lübke et al., 1997; Deller et al., 1999; Takács et al., 2008). Thus, medial septal cells (both excitatory cholinergic and inhibitory GABAergic subtypes) appear ideally organized to regulate translamellar inhibition in the dentate gyrus by influencing mossy cells and inhibitory interneurons.

Perhaps most importantly, the medial septal projections to the hippocampus are topographically organized (Amaral and Kurz, 1985; Kiss et al., 1990), suggesting that lamellar information within the hippocampus is conveyed topographically to cells in the medial septum via the somatostatin-positive interneurons (because these cells receive lamellar input from the mossy fibers) and, in return, medial septal cells control basket cells and mossy cells in particular lamellae via topographical projections back to the hippocampus. *Therefore, we hypothesize that medial septal GABAergic neurons, by topographically inhibiting basket cells within a given lamella, convert perhaps multi-lamellar excitatory inputs from the entorhinal cortex into signals that selectively activate individually disinhibited lamellae*. By disinhibiting one granule cell layer “lamella” via inhibition of its basket cells (Bilkey and Goddard, 1987), medial septal GABAergic cells may establish lamellar function in the dentate gyrus without requiring that the entorhinal input to the dentate gyrus be as “lamellar” as the mossy fiber pathway. That is, *a divergent afferent pathway need not be viewed as being antithetical to the concept of lamellar function because a highly focused topographic inhibitory input to basket cells in one lamella could result in a highly restricted ‘lamellar’ response to a divergent excitatory input from the entorhinal cortex*. This control mechanism could be extraordinarily precise depending on the specificity of the topographical septal input to inhibitory interneurons in both the longitudinal and transverse planes. The hypothesis that the medial septum might regulate longitudinal transmission may be consistent with the conclusions of recent studies indicating that “theta waves” travel throughout the longitudinal hippocampal axis (Lubenov and Siapas, 2009; Patel et al., 2012).

A similar external (extrahippocampal) control mechanism may be exerted by the serotonergic input from the median raphe nucleus (Winson, 1980; Nitz and McNaughton, 1999), which selectively innervates several interneuron populations, but specifically avoids parvalbumin-positive basket cells that mediate axo-somatic inhibition (Halasy et al., 1992; Acsády et al., 1993). Thus, input from the brainstem median raphe nucleus may selectively influence several interneuron subpopulations that receive lamellar input from granule cells and convey that information to medial septal cells (Zappone and Sloviter, 2001) and other targets. In this way, the serotonergic input might influence lamellar function by modulating the inhibitory output of the somatostatin-positive HS cells, whereas the cholinergic and GABAergic medial septal cells may modulate lamellar function by topographically exciting or inhibiting mossy cells and basket cells. Under this scenario, external inputs to the hippocampus from the septum, brainstem, hypothalamus, and thalamus (Amaral and Lavenex, 2007) could conceivably impose “lamellar” function on a structure with both lamellar and longitudinal structural components, and serve as a switching mechanism that shifts the hippocampus from a “lamellar” to a “less lamellar-” or “non-lamellar” mode of function. Incorporating these features of hippocampal organization into the discussion brings an entirely different perspective to the assertion that the simple existence of any longitudinal pathways should be viewed as being inherently antithetical to the concept of lamellar function (Amaral and Witter, 1989).

## On the Possibility of Lamellar and Non-Lamellar Modes of Hippocampal Function

It seems at least hypothetically conceivable that the hippocampus might normally alternate between “lamellar” and “non-lamellar” functional modes during different behavioral states, e.g., awake function vs. dreaming. A fully lamellar functional mode may be necessary during awake interaction with the real spatial and cognitive world, when mistakes in memory or spatial localization have severe consequences, whereas a non-lamellar mode of function may be operative during dreaming, when consequence-free interactions with the “intracranial” virtual version of the real world serve useful purposes, and when memory recall is impaired (De Gennaro et al., 2010). This hypothesized dual function for the hippocampus may be analogous to the dual function of the thalamus, which shifts from a topographically faithful sensory relay mode to a non-topographical rhythmic mode depending on the behavioral state and the activity of external ascending or descending influences (Steriade et al., 1969; McCormick and von Krosigk, 1992).

The obvious advantage of duality in hippocampal function lies in having one piece of hardware serving multiple purposes depending on the behavioral state, or running different “programs” or “spatial maps” under different conditional states. Because mossy cells innervate both granule cells and inhibitory interneurons (Sloviter et al., 2003), the granule cell layer might shift between lamellar- and non-lamellar modes of function depending on whether granule cell associative excitation- or translamellar lateral inhibition predominates (Sloviter et al., 2012). That is, when mossy cell excitation of granule cells and basket cells results in net inhibition of granule cells, lamellar function might be operative. Conversely, under unknown conditions in which mossy cells might predominantly excite granule cells, i.e., if and when basket cells and axo-axonic cells are powerfully inhibited, perhaps via septal input as described above, or via basket cell–basket cell interactions (Bartos et al., 2001), the granule cell layer might theoretically respond to entorhinal excitation as a functional “syncytium,” with the hippocampus operating as a single giant cortical module (Wittner et al., 2007).

Just as “petit mal” seizures apparently involve a defective switching of the thalamic pacemaker that causes sleep-like rhythms to occur during the awake state (Beenhakker and Huguenard, 2009), a failure to switch correctly between lamellar and non-lamellar hippocampal states might give rise to psychotic ideation, which can occur during spontaneous temporal lobe seizures (Nadkarni et al., 2007; Elliott et al., 2009; Small et al., 2011). A complete or partial shift from a “lamellar” to a “non-lamellar” functional state (Paré and Llinás, 1994) could occur under different normal behavioral conditions (Winson and Abzug, 1977; Lapray et al., 2012), or in temporal lobe epilepsy, where the injury-induced loss of mossy cells may cause normally functionally separated granule cells to become disinhibited, to coalesce, and to discharge synchronously in response to entorhinal input (Sloviter et al., 2012). Lamellar malfunction could therefore theoretically underlie both psychiatric and epileptic phenomena in which the hippocampus is causally involved. If mossy cells do indeed establish pattern separation in the dentate gyrus (Jinde et al., in press), then any genetic or developmental abnormalities that affect the final innervation patterns that mossy cells form postnatally might produce abnormal lateral inhibitory “maps” that influence lamellar hippocampal function and underlie some psychiatric and/or neurological disorders (Small et al., 2011), although this is admittedly speculative.

## How Wide is a “Lamella?”

Although few disagreements exist about any specific features of hippocampal anatomy, perspectives differ with regard to their possible physiological implications because each perspective is dependent on how the anatomical data are judged, included, excluded, weighted, and related to the physiology. For example, it is difficult to address the question of how “wide” a lamella might be because we view the maximum lengths of longitudinally projecting axons to be only superficially related to the parameters of lamellar physiology. In addition, the fact that very few granule cells discharge at any given time (Jung and McNaughton, 1993; Alme et al., 2010), and the observation that individual granule cells form non-convergent connections with a small number of CA3 pyramidal cells (Acsády et al., 1998), contrast with the simplistic idea that entorhinal input causes the discharge of all granule cells and CA3 pyramidal cells within a lamella. Thus, it should be recognized that a perforant path stimulus delivered in the laboratory, which massively excites many targeted granule cells, is likely to be far less selective than a perforant path volley that occurs physiologically in a freely moving animal. In addition, lamellar function may have significant transverse, as well as longitudinal, components. That is, some granule cells within a transverse lamella might selectively innervate a subpopulation of CA3a pyramidal cells, whereas other granule cells within the same lamella might preferentially target a subset of CA3b pyramidal cells (Deguchi et al., 2011; Moser, 2011). If this is the case, the discharges of individual granule cells within the same transverse plane could encode different memories or locations (Alme et al., 2010) based on differences in the targeting of each granule cell. Therefore, physiological pattern separation may occur on multiple levels (multiple locations and long both the longitudinal and transverse axes) in a far more complex manner than anything that can be adequately described by citing a particular anatomical distance along the septo-hippocampal axis. For these reasons, we hesitate to speculate further on the possibly irrelevant and unanswerable question of how “wide” a lamella might be, or how many “lamellae” a hippocampus might contain. However, the observations that both dentate mossy cells and CA3 pyramidal cells innervate their longitudinally distant targets unevenly, with periodicities of ∼900 μm for mossy cells (Soltesz et al., 1993) and ∼300–600 μm for CA3 pyramidal cells (Hsu and Buzsáki, 1993; Li et al., 1994), may be particularly relevant in this regard.

## Testing the Hypotheses

The value of any hypothesis depends largely on how testable and falsifiable it is (Popper, 1959; Kuhn, 1962). We contend that many of the hypotheses we have addressed or suggested in this review are eminently testable. It is certainly feasible to describe the topographical details of the entorhinal and septal inputs to the hippocampus more definitively, and it is similarly possible to conduct *in vivo* studies to identify the conditions under which longitudinal pathways have net excitatory or inhibitory effects on distant principal cell excitability. The septal inputs to hippocampal principal cells and interneurons can be elucidated in terms of their topography in both the longitudinal and transverse axes, and selective elimination of septal and other neurons, as has been done successfully in the hippocampus (Martin and Sloviter, 2001), is a practical approach that might clarify the role of extrahippocampal inputs in hippocampal network function. Whether it is currently practical and feasible to definitively demonstrate dual modes of lamellar hippocampal function is unclear, but one purpose in raising these issues is to emphasize that a reconsideration of the possibilities will suggest practical strategies to others and facilitate alternate interpretations of results obtained in behavioral and electrophysiological studies. As always, a main obstacle to progress is the belief that the important issues have been “settled” and, therefore, continued discussion is unnecessary and maybe even detrimental to further advances in understanding. Our main point is that the structural and functional organization of the hippocampal formation is still incompletely understood, and that all possibilities should be considered undogmatically when the results of experiments are interpreted, or when the hippocampus is modeled under a particular set of assumptions.

## Summary and Conclusion

If the lamellar hypothesis is strictly defined from an anatomical perspective to imply that all excitatory activity must be restricted to single transverse slices no wider than the mossy fiber pathway, then even a single axon traveling outside a thin transverse hippocampal “slice” might refute the entire hypothesis. In our view, there is little to be gained from such a restrictive definition of lamellar organization, which was neither stated nor implied in the original or subsequent discussions of the hypothesis (Andersen et al., 1971, 2000; Lømo, 1971, 2009). We suggest that modification of the original lamellar hypothesis to incorporate new data and evolving perspectives is the prudent course, and preferable to outright rejection of an unnecessarily strict interpretation of the hypothesis. With that in mind, we suggest that lamellar organization is a likely design feature of the dentate gyrus in particular, and that the mossy cell-derived longitudinal fiber system in the dentate gyrus may subserve that function by mediating lateral inhibition (as well as underlying associative excitation) in the granule cell layer (Sloviter, 1994; Sloviter and Brisman, 1995; Zappone and Sloviter, 2004; Jinde et al., in press). Once the lamellar impulses reach the CA3 region via the mossy fiber pathway, recurrent excitatory connections between pyramidal cells (Miles and Wong, 1987), and the extensive transverse and longitudinal axons of CA3 pyramidal cells, presumably convey this lamellar information topographically to multiple targets, including inhibitory interneurons and CA1 pyramidal cells (Ishizuka et al., 1990; Tamamaki and Nojyo, 1991; Li et al., 1994; Wittner et al., 2006), which finally activate the subiculum and entorhinal areas topographically (Amaral and Lavenex, 2007) for purposes that we do not yet fully understand.

Based on the sum of the available anatomical information, and the stipulation that every general anatomical statement is a generalization for which exceptions can be made, we suggest the following perspective. First, there are significant similarities between dentate granule cells, the main “entrance gate” to the hippocampus (Winson and Abzug, 1977), and CA1 pyramidal cells, the main output population. Granule cells and CA1 pyramidal cells lack recurrent excitatory connections, and their axons form few commissural or associational collaterals (Amaral and Lavenex, 2007). Therefore, both granule cells and CA1 pyramidal cells appear designed to receive and faithfully transmit “lamellar” information to their specific targets, which are the area CA3 neurons and the subiculum, respectively. Second, the lamellar granule cell output excites CA3 pyramidal cells, which uniquely form recurrent excitatory connections with other CA3 pyramidal cells, and also innervate CA1 pyramidal cells (Ishizuka et al., 1990; Li et al., 1994), axo-somatic interneurons in particular (Wittner et al., 2006), and targets in the lateral septum (Swanson et al., 1981). If normal hippocampal function involves synchronized excitation of spatially separated CA1 pyramidal cells (Deadwyler and Hampson, 1999; Hampson et al., 1999; Senior et al., 2008), then longitudinal associational axons of CA3 pyramidal cells would presumably be required for lamellar function, rather than being antithetical to it.

The scenarios discussed above emphasize our clearly subjective judgments about which anatomical features might be functionally most important, and we have not highlighted other anatomical features, such as the mossy fibers that turn longitudinally at the end of their termination in area CA3a (Swanson et al., 1981; Tamamaki and Nojyo, 1991; Acsády et al., 1998; Ropireddy and Ascoli, 2011), the entorhinal input from Layer III that innervates stratum lacunosum-moleculare interneurons and the distal apical dendrites of CA1 pyramidal cells (Jones, 1993; Vinogradova, 2001), semilunar granule cells (Hyde and Strowbridge, 2012), the perforant path fibers that directly innervate the distal apical dendrites of CA3a and CA3b pyramidal cells, or the CA3c-mossy cell “back-projection” (Amaral and Lavenex, 2007). Neurons send axons in response to molecular guidance cues presumably related to concentration gradients (Kolodkin and Tessier-Lavigne, 2011), and it is possible that some minor anatomical features, such as the longitudinal turn of mossy fibers at the end of their trajectory, or the CA3c-mossy cell “back-projection,” could be functionally insignificant developmental aberrations that unavoidably develop in response to nearby attractant gradients.

We do not pretend to know which of these and other anatomical features may have major, minor, or negligible functional importance, or how, and to what extent, each anatomical feature dictates or influences hippocampal encoding of memories, or creation of maps of the spatial environment (Vinogradova, 2001; Giocomo et al., 2011; Krupic et al., 2012). Multiple hypotheses exist regarding the relationship between the dentate gyrus and area CA3 in memory formation and storage (McNaughton and Morris, 1987; McNaughton, 1989; Treves and Rolls, 1992; Patton and McNaughton, 1995; Colgin et al., 2008; Rolls, 2010), and it is beyond the scope of this work to suggest a comprehensive model of hippocampal function that incorporates and assigns relative importance to every known structural and molecular feature. Here we are simply arguing in favor of being open to multiple possibilities, and being willing to consider all of the relevant data, regardless of whether they support or undermine a particular perspective. In addition, it is at least possible that studying anesthetized animals and tissue slices gives us an inherently inaccurate picture of hippocampal physiology in its natural state. From our perspective, the sum of the available data favors the view that: (1) the existence of excitatory, longitudinally projecting associational pathways of dentate mossy cells and CA3 pyramidal cells is in no way antithetical to the concept of lamellar function; it depends on what these pathways do, and; (2) that afferent activity from the septum and brainstem nuclei (Bland and Oddie, 1998), which topographically target mossy cells and inhibitory interneurons, might play a central role in defining lamellar function, and perhaps switching between “lamellar” and “non-lamellar” functional states.

Clearly, inputs from extrahippocampal brain regions, and other factors, including the spatio-temporal interactions between principal cells (Senior et al., 2008) and among inhibitory neuron subpopulations (Bartos et al., 2001) and principal cells (Klausberger and Somogyi, 2008; Lapray et al., 2012), may play crucial roles in establishing and regulating lamellar function. The recent finding that there may be parallel, target-specific “subpathways” within each trisynaptic circuit (Deguchi et al., 2011; Moser, 2011), which is suggestive of an even more “lamellar” functional separation of signals in the dentate gyrus than originally conceived by Andersen et al. (1971), emphasizes the importance of recognizing that all “pathways” or influences are not anatomically obvious, and therefore, it might be prudent to have a malleable definition of what constitutes, defines, and governs “lamellar” function. An awareness that current hypotheses need to leave room for new processes and principles of organization that are yet to be discovered might facilitate reaching a closer approximation of the “truth,” whatever that might someday be understood to be.

## Conflict of Interest Statement

The authors declare that the research was conducted in the absence of any commercial or financial relationships that could be construed as a potential conflict of interest.
